# Association of Common Polymorphisms in *TNFA*, *NFkB1* and *NFKBIA* with Risk and Prognosis of Esophageal Squamous Cell Carcinoma

**DOI:** 10.1371/journal.pone.0081999

**Published:** 2013-12-04

**Authors:** Meenakshi Umar, Rohit Upadhyay, Shaleen Kumar, Uday Chand Ghoshal, Balraj Mittal

**Affiliations:** 1 Department of Genetics, Sanjay Gandhi Post Graduate Institute of Medical Sciences, Lucknow, Uttar Pradesh, India; 2 Department of Radiotherapy, Sanjay Gandhi Post Graduate Institute of Medical Sciences, Lucknow, Uttar Pradesh, India; 3 Department of Gastroenterology, Sanjay Gandhi Post Graduate Institute of Medical Sciences, Lucknow, Uttar Pradesh, India; Winship Cancer Institute of Emory University, United States of America

## Abstract

**Background:**

Tumour necrosis factor-alpha (TNF-α) and nuclear factor of kappa light chain gene enhancer in activated B cells (NF-κB) play critical role in carcinogenesis processes like tumour initiation, proliferation, migration and invasion. Single nucleotide polymorphisms in TNF-α, NF-κB and its inhibitor IκB genes were shown to be associated with susceptibility and prognosis of several cancers; however, their role in esophageal squamous cell carcinoma (ESCC) is not well recognised. Therefore, in present study, we aimed to investigate association of common polymorphisms in TNFA, NFkB1 and NFKBIA with risk and prognosis of ESCC in northern Indian population.

**Methods:**

We genotyped 290 ESCC patients (including 162 followed up cases) and 311 mean age, gender and ethnicity matched controls for TNFA -308G>A, *NFkB1* -94ATTG ins/del and *NFKBIA* (-826C>T and 3’UTRA>G) polymorphisms using PCR alone or followed by RFLP and TaqMan assay.

**Results:**

*TNFA* -308GA genotype was associated with increased risk of ESCC specifically in females and in patients with regional lymph node involvement, while, NFKBIA -826CT+TT genotype conferred decreased risk of ESCC in females. Haplotypes of NFKBIA -826C>T and 3’UTRA>G polymorphisms, C_-826_G_3_’_UTR_ and T_-826_A_3_’_UTR_, were associated with reduced risk of ESCC. No independent role of *NFkB1* -94ATTG ins/del polymorphism in susceptibility of ESCC was found. Multi-dimensionality reduction analysis showed three factor model *TNFA*-308, *NFKBIA*-826, *NFKBIA* 3’UTR as better predictor for risk of ESCC. Furthermore, combined risk genotype analysis of all studied polymorphisms showed increased risk of ESCC in patients with 1-3 risk genotype compared to ‘0’ risk genotype. Survival analysis did not show any significant prognostic effect of studied polymorphisms. However, in stepwise multivariate analysis, metastasis was found to be independent prognostic predictor of ESCC patients.

**Conclusion:**

*TNFA*-308 and *NFKBIA* (-826C>T and 3’UTRA>G) polymorphisms may play role in susceptibility but not in prognosis of ESCC patients in northern Indian population.

## Introduction

Chronic inflammation, a critical component of tumour microenvironment, is involved in pathogenesis of approximately 25% of all human cancers including esophageal cancer (EC) [1,2]. Tumour necrosis factor-alpha (TNF-α) and nuclear factor of kappa light chain gene enhancer in activated B cells (NF-κB) are two major mediators of inflammation in cancer and they are intricately linked to malignant processes like tumour initiation, proliferation, invasion and angiogenesis [3,4]. 

TNF-α gene (*TNFA*) spans about 3 kilo-base pair on chromosome 6p21.3 and contains 4 exons. It encodes a pro-inflammatory pleiotropic cytokine that plays role in cell differentiation, proliferation, apoptosis and immunity [5]. Deregulation of TNF-α is implicated in wide spectrum of diseases like diabetes, osteoporosis, autoimmune diseases and cancers [6]. Also, expression of TNF-α was found to progressively increase from precancerous to cancerous lesions of esophageal carcinoma pointing its critical role in EC [7]. Several functional polymorphisms are present in the promoter region of *TNFA*; however the most documented single nucleotide polymorphism (SNP) is located at 308 nucleotide position resulting in substitution of G to A (rs1800629). Epidemiological studies exploring association of *TNFA*-308 G>A polymorphism with cancer risk are inconsistent. While, some studies have shown increased risk of carcinoma of breast [8], colon [9], oral cavity [10] and cervix [11], other studies did not find any role of the polymorphism in cervical [12] and prostate cancers [13]. In esophageal cancer, there are two reports which failed to find any association of the polymorphism [14,15]. *TNFA*-308 polymorphism may also have prognostic implication as it was found to confer adverse outcome to head & neck cancer and gastro-esophageal patients [15,16]. 

NF-κB is a family of transcription factors that are activated by TNF-α in classical canonical pathway [17]. There are five members of NF-κB: RelA, RelB, c-Rel, p50/105 (encoded by *NFKB1* gene; chromosomal location: 4q23-q24), and p52/p100. The dimeric form of NF-κB, p50/RelA, is the most common form [18]. In un-stimulated cell, NF-κB remains sequestered in cytoplasm by its inhibitor IκB. Following activating stimuli, IκBs are phosphorylated and degraded, so NF-κB is activated and is translocated to the nucleus to initiate the target gene expression [19]. The IκB family also constitutes several members among which IκBα (encoded by *NFKBIA*, located on 14q13) is classical form that can be found in the cytoplasm and nuclei [20]. NF-κB was reported to be constitutively activated in esophageal squamous cell carcinoma (ESCC) tissues and ESCC cell lines (Eca109 and EC9706) [21,22]. While, a study has also shown amplification and overexpression of *NFKBIA* gene in KYSE series EC cell lines [23]. Several polymorphisms are present in *NFKB1* (1900 SNPs) and *NFKBIA* (158 SNPs) according to dbSNP database (www.ncbi.nlm.nih.gov/snp), however, previous studies have extensively explored role of common polymorphic variants in promoter region of *NFKB1* (-94 ATTG ins/del; rs28720239) and *NFKBIA* (-826 C>T; rs2233406) and 3’UTR region of *NFKBIA* (3’UTR A>G; rs696) in susceptibility and prognosis of various cancers [24–29]. Literature exploring association of these *NFKB1* and *NFKBIA* variants in EC are missing till now. So, in the present study, we investigated the association of TNFA-308 G>A, *NFKB1* -94ATTG ins/del and *NFKBIA* (-826 C>T and 3’UTR A>G) polymorphisms with susceptibility to esophageal squamous cell carcinoma (ESCC) or its clinical phenotypes, their interaction with environmental risk factors and their role in survival outcome of ESCC patients.

## Materials and Methods

### Ethics statement

The study was approved by the ethical committee of Sanjay Gandhi Postgraduate Institute of Medical Sciences, Lucknow (28^th^ Sanjay Gandhi Post Graduate Institute of Medical Sciences Ethics Committee Meeting on May 14, 2003) and written informed consent was obtained from all recruited individuals. All the clinical and demographical details were recorded anonymously by one individual (data collector), while experiments and analysis was performed by another individual (investigator). The study subjects were coded in random numbers and identity of patients (name and their hospital identity number) was never revealed to investigator at any stage of the study. Blood samples and other details from all study subjects were collected after taking their informed written consent.

### Subject recruitment

During the period of 7 years from 2005 to 2012, 311 EC cases were recruited from out-patients clinic of Gastroenterology and Radiotherapy departments, Sanjay Gandhi Post Graduate Institute of Medical Sciences, Lucknow. The diagnosis of patient was confirmed by pathologist. EC patients with two major histopathologies: squamous cell carcinoma (SCC) and adenocarcinoma (ADC) were recruited in the study. However, the frequency of ADC cases (6.8%) was low in our study and etiologies of SCC and ADC are entirely different; therefore, we carried our analysis in 290 incident ESCC cases only. Patients with pre-malignant conditions like corrosive esophageal injury, Achalasia injury, Barrett’s esophagus or Gastroesophageal reflux diseass and prior treatment before enrolment were excluded from the study. Data on demography, clinical parameters (like tumour location and regional lymph node involvements) and environmental exposures (alcohol consumption, smoking of cigarette or ‘bidi’ and smokeless tobacco usage) were recorded only for the patients in a standard proforma from medical records and personal interview as described previously [30]. During the same time frame, 311 mean age, gender and ethnicity matched cancer free controls were recruited in the study. The exclusion criteria of controls were presence of any malignancy, chronic disease or pre-malignant conditions.

For survival analysis, patients who had received radiotherapy (RT) with or without concurrent cisplatin based chemotherapy (CT) were followed up during 2005-2012. Many surgically resected cases and patients who discontinued their treatment could not be followed up, so prognostic evaluation was limited to 162 cases. The inclusion criteria of patients for survival analysis was patients who had received RT based treatment regime i.e. patients who had received RT or RT along with CT. However, patients who were referred for surgery or for palliative treatment or with previous history of surgical resection were excluded in the survival analysis. The maximum follow up time was 72.6 months and the median follow up time was 9.3 months. Dosage of RT and CT, dysphagia grade and duration, tumour stage, Karnofsky performance score and other parameters were recorded for each followed patients. The patients were prospectively followed up every six months from the time of enrolment through telephonic conversations or their visit to out-patient clinic until death or end of the study. The regional lymph node involvement was recorded as ‘present’ or ‘absent’. Around, 47% (120/255) of cases had regional lymph node involvement in overall-group of patients, while, in followed up patients group, regional lymph node positivity was recorded in only 39.5% (64/162) of cases.

### DNA extraction and SNP genotyping

A 3 ml venous blood was collected from each subjects in sterile EDTA vacutainer tubes and stored at -40°C until DNA extraction. Genomic DNA was extracted from peripheral blood leukocytes using standard salting out method [31]. The genotyping of *TNFA*-308 G>A and *NFKB1 -*94ins/del ATTG polymorphisms were carried out through PCR based methods using primer sequences as described previously [32,33]. NFKBIA -826 C>T and 3’UTR A>G polymorphisms were genotyped by TaqMan assay and PCR RFLP [34] respectively. The details of genotyping methods are described in Table S1. To improve genotyping quality and validation, 20% of samples were re-genotyped by other laboratory personnel and genotyping results were reproducible with no discrepancy. Lab personals were also blinded to case control status during genotyping to eliminate bias.

### Statistical analysis

The effective sample size for case-control study was calculated using Quanto 1.1 ver. software [35], while minimum sample size for case-only survival analysis for obtaining 80% power was estimated using PS version 2.1.3.1 software [36]. Genotypic frequencies of each studied polymorphisms among control subjects were checked for Hardy-Weinberg equilibrium (HWE) using goodness-of-fit χ^2^ test. Binary logistic regression was applied to calculate Odds ratio (OR) and 95% Class interval (CI) for various predictors after adjustment of covariates like age and gender. Haplotype analysis of *NFKBIA* polymorphisms was conducted using SNPAnalyzer version 1.0 [37]. Since response rate was low, case only analysis was performed for gene-environment interaction. In case of multiple comparisons, False discovery rate (FDR) test was applied to avoid type 1 error and the threshold value was taken as 0.10. Multi-factor Dimensionality Reduction (MDR) analysis was performed to evaluate the high order interaction between the polymorphisms using MDR 3.0.2 software (www.multifactordimensionalityreduction.org). MDR software gives number of output parameters like cross validation consistency (CVC), testing accuracy (TA), balanced training accuracy for different interactions and single best model is identified as interaction that had maximum CVC and TA. Statistical significance of the model was evaluated using a 1000-fold permutation test. Kaplan Meier and Log rank tests were carried out to estimate the difference in survival times according to genotypes and clinical/demographical characteristics. Survival time was calculated from date of ESCC diagnosis to death of patients or date of last follow up. Univariate Cox regression analysis was done to determine predictive factor of ESCC survival by estimating Hazard ratio (HRs) and 95% CI. Multivariate analysis was also performed, in which all variables were first entered together in single step and after that in stepwise manner also, to identify independent prognostic predictor of ESCC. Two models (forward selection and backward elimination) were employed in stepwise Cox regression analysis. All statistical analyses were performed with SPSS software version 15.0 (SPSS, Chicago, Illinos, USA) and differences were taken as significant when two sided P-value was less than 0.05.

## Results

The power calculation analysis showed that at minimum minor allelic frequency (MAF) of 5.4% (as reported for Gujarati Indian population in Hapmap database for *TNFA*-308 G>A polymorphism) and genetic effect of 2.0, the case control pair of 240 was sufficient to achieve 80% power. 

### Characteristics of subjects included in susceptibility analysis

The demographic and clinical characteristics of cases and control are shown in Table 1. There was no significant difference in median age and gender distribution between cases (Median age = 57 years and Males: 72.8%) and controls (Median age: 55 years and Males: 71.1%). Majority of patients (59.3%) had tumour at middle third location and 47% of cases had regional lymph node involvement. Data on environmental risk factors showed that 79% of cases used tobacco in some form (chewing, smoking or snuff) and 28.5% had alcohol drinking habit. 

**Table 1:** Demographic and clinical characteristics of study subjects.

**Variables**	**N (%)**
**Esophageal Squamous Cell Carcinoma (ESCC**)** patients**	290
Median age	57 years
**Gender**	
Males	211 (72.8)
Females	79 (27.2)
Ethnicity	northern Indian
**Controls**	311
Median age	55 years
**Gender**	
Males	221 (71.1)
Females	90 (28.9)
Ethnicity	northern Indian
Tumour location*	
Upper	45 (15.5)
Middle	172 (59.3)
Lower	73 (25.2)
Regional Lymph node*	
Present	120 (47.1)
Absent	135 (52.9)
Environmental exposures of ESCC patients^*^	
**Tobacco habit**	
Smokers	45 (16.0)
Tobacco chewers	90 (31.9)
Smokers+ tobacco chewers	89 (31.6)
Non-tobacco users	58 (20.6)
**Alcohol habit**	
Drinkers	79 (28.5)
Non-drinker	198 (71.5)

^*^ Data was missing in some cases

### Characteristics of subjects included in survival analysis

In group of followed up patients, 29.6% (48/162) of patients had received RT only, while, 70.4% (114/162) of cases had received both CT and RT. The ratio of alive, dead and lost to follow up cases was 25 (15.4%): 76 (46.9%): 61 (37.7%). Most of the patients (71.9%) had tumour either in T1 or T2 stage. Median dysphagia duration was 3 month. The frequency of dysphagia grade 1+2, 3 and 4 was 99 (61.5%), 49 (30.4%) and 13 (8.1%) respectively. Also, majority of patients were tobacco users (77.8%), 48.7% of cases were smokers, 29.9% were alcohol drinkers and 23.5% of patients had occupational exposure in the followed up group. 

### Association of the TNFA-308 G>A, *NFKB1* -94ATTG ins/del and *NFKBIA* (-826 C>T and 3’ UTR A>G) polymorphisms with overall risk of ESCC

Chi square test showed genotypic frequencies of polymorphisms in *TNFA*, *NFKB1* and *NFKBIA* were in accordance with HWE in controls (P>0.05 in each case). When genotypic distribution of *TNFA* -308 G>A polymorphism was compared between cases and controls, 1.7 fold increased risk of ESCC was observed with *TNFA* -308 GA genotype compared to GG genotype (OR = 1.73, 95% CI = 1.13-2.67, P = 0.013). Also, *TNFA* -308 A allele was associated with enhanced risk of ESCC compared to G allele (OR = 1.62, 95% CI = 1.08-2.42, P = 0.019) (Table 2). No associations of *NFKB1* -94 ins/del, and *NFKBIA* (-826 C>T and 3’UTR A>G) polymorphisms were observed with the overall risk of ESCC. 

**Table 2:** Frequency distribution and association of the selected polymorphisms with risk of esophageal squamous cell carcinoma (ESCC).

**Genotypes/alleles**	**Controls N (%)**	**ESCC Patient N (%)**	OR*(95%CI) P value	**P for trend**
***TNFA*-308 G>A polymorphism**				
GG	268 (86.2)	227 (78.3)	Reference	**0.013**
GA	42 (13.5)	62 (21.4)	**1.73 (1.13-2.67) 0.013**	
AA	1 (0.3)	1 (0.3)	1.16 (0.07-18.63) 0.918	
GA+AA	43 (13.8)	63 (21.7)	**1.72 (1.12-2.64) 0.013**	
*G* allele	578 (92.9)	516 (89.0)	Reference	
*A* allele	44 (7.1)	64 (11.0)	**1.62 (1.08-2.42) 0.019**	
***NFKB1* -94ATTG ins/del polymorphism**				
ATTG_1/_ATTG_1_	22 (7.1)	27 (9.3)	Reference	0.101
ATTG_1_/ATTG_2_	129 (41.5)	132 (45.5)	0.85 (0.46-1.57) 0.598	
ATTG_2_/ATTG_2_	160 (51.4)	131 (45.2)	0.68 (0.37-1.25) 0.217	
ATTG_1_/ATTG_2_+ ATTG_2_/ATTG_2_	289 (92.9)	263 (90.7)	0.76 (0.42-1.36) 0.349	
ATTG_1_ allele	173 (27.8)	186 (32.1)	Reference	
ATTG_2_ allele	449 (72.2)	394 (67.9)	0.82 (0.64-1.05) 0.122	
**NFKBIA -826 C>T polymorphism**				
CC	149 (47.9)	145 (50.0)	Reference	0.858
CT	141 (45.3)	122 (42.1)	0.89 (0.63-1.24) 0.472	
TT	21 (6.8)	23 (7.9)	1.14 (0.60-2.15) 0.691	
CT+TT	162 (52.1)	145 (50.0)	0.92 (0.67-1.26) 0.598	
C allele	439 (70.6)	412 (70.6)	Reference	
T allele	183 (29.4)	168 (29.0)	0.98 (0.76-1.26) 0.866	
***NFKBIA* 3’UTR A>G polymorphism**				
AA	59 (19.0)	71 (24.5)	Reference	0.274
AG	165 (53.1)	140 (48.3)	0.69 (0.45-1.04) 0.077	
GG	87 (28.0)	79 (27.2)	0.73 (0.46-1.17) 0.190	
AG+GG	252 (81.0)	219 (75.5)	0.70 (0.48-1.04) 0.079	
A allele	283 (45.5)	282 (48.6)	Reference	
G allele	339 (54.5)	298 (51.4)	0.87 (0.69-1.09) 0.236	

*NFKB1* -94ATTG_1_ allele stands for deletion allele and ATTG_2_ stands for insertion allele^*^ age and gender adjusted odds ratio; significant values are shown in bold;

### Survival analysis

Univariate and multivariate Cox regression analysis showed no significant effect of TNFA-308 G>A, *NFKB1* -94ATTG ins/del, *NFKBIA* (-826 C>T and 3’UTR A>G) polymorphisms on the survival outcome of ESCC patients (Table 3 and 4). However, when all of the variables in the study (genetic polymorphisms and clinico-pathological variables) were entered in single step in multivariate analysis, presence of metastasis conferred poor survival outcome to ESCC patients (HR = 3.39, 95% CI = 1.44-7.98, P = 0.005). Similarly, stepwise regression analysis (in both forward selection and backward elimination model) indicated metastasis as an independent predictor of prognosis of ESCC patients (HR = 2.72, 95% CI =1.44-5.14, P = 0.002) (Table 4).

**Table 3:** Univariate survival analysis of selected gene polymorphisms in ESCC.

**Genotypes**	**N (%)**	**Median survival (in months)**	**Log Rank P value**	**HR (95% CI) P value**
***TNFA* -308 G>A polymorphism**				
GG	126 (77.8)	16.73	0.368	Reference
GA	35 (21.6)	22.94		0.77 (0.44-1.34) 0.347
AA	1 (0.6)	9.26		3.42 (0.45-25.99)0.235
GA+AA	36 (22.2)	15.00	0.503	0.80 (0.47-1.39) 0.434
***NFKB1* -94 ATTG ins/ del polymorphism**				
ATTG_1/_ATTG_1_	15 (9.3)	27.33	0.923	Reference
ATTG_1_/ATTG_2_	76 (46.9)	20.80		0.93 (0.42-2.04) 0.856
ATTG_2_/ATTG_2_	71 (43.8)	15.60		1.00 (0.46-2.19) 0.999
ATTG_1_/ATTG_2_+ ATTG_2_/ATTG_2_	147 (90.7)	16.73	0.875	0.96 (0.46-2.03) 0.923
**NFKBIA -826 C>T polymorphism**				
CC	83 (51.2)	16.73	0.617	Reference
CT	68 (42.0)	18.80		0.81 (0.50-1.32) 0.397
TT	11 (6.8)	10.67		1.27 (0.53-3.05) 0.598
CT+TT	79 (48.8)	18.50	0.644	0.87 (0.55-1.37) 0.546
***NFKBIA 3’UTR A>G* polymorphism**				
AA	41 (25.3)	14.60	0.837	Reference
AG	79 (48.8)	20.80		1.00 (0.57-1.76) 0.992
GG	42 (25.9)	11.60		1.18 (0.62-2.23) 0.621
AG+GG	121 (74.7)	18.50	0.865	1.05 (0.62-1.80) 0.846

HR: hazard ratio

**Table 4:** Multivariate analysis of various clinical parameters and inflammatory gene polymorphisms.

**Variables**	**Hazard ratio**	**95% Class Interval**	**P value**
**Enter method (When all variables were entered in single step)**			
**Age**	0.98	0.96- 1.01	0.155
**Sex**	0.70	0.28-1.75	0.444
**Dysphagia Grade**			
Grade 1+2 vs. 3	0.64	0.32-1.28	0.208
Grade 1+2 vs. 4	0.64	0.13-3.31	0.598
**Dysphagia duration**	0.95	0.88-1.04	0.262
**Regional nodal status** (Presence vs. absence)	1.14	0.63-2.08	0.658
**Tobacco usage** (Users vs. non-users)	2.05	0.75-5.62	0.161
**Smoking** (Smokers vs. non-smokers)	0.85	0.41-1.76	0.662
**Alcohol drinking** (Drinkers vs. non-drinkers)	0.84	0.43-1.66	0.623
**Occupational Exposure** (Yes vs. no)	2.06	0.95-4.49	0.069
**Tumour length**	0.99	0.87-1.11	0.824
**Tumour staging** (T1+T2 vs. T3)	1.21	0.56-2.61	0.636
**Metastasis** (Yes vs. No)	**3.39**	**1.44-7.98**	**0.005**
**Radiotherapy dosage** ( ≤ 50Gy vs. >50Gy)	1.18	0.57-2.45	0.656
**Chemotherapy given** (No vs. Yes)	1.06	0.51-2.20	0.884
			***TNFA*-308 G>A polymorphism**
GG vs. GA	0.85	0.25-2.85	0.786
GG vs. AA	2.15	0.15-30.38	0.571
			***NFKB1* -94ATTG ins/del polymorphism**
ATTG_1_/ATTG_2_ vs. ATTG_1_/ATTG1	1.45	0.43-4.87	0.544
ATTG2/ATTG2 vs. ATTG1/ATTG1	1.76	0.51-6.04	0.368
			**NFKBIA -826 C>T polymorphism**
CT vs. CC	0.81	0.40-1.62	0.544
TT vs. CC	1.34	0.34-5.26	0.674
			***NFKBIA* 3’UTR A>G polymorphism**
AG vs. AA	1.05	0.35-3.17	0.932
GG vs. AA	1.50	0.46-4.88	0.502
**Dichotomized risk genotype (1-3 vs. 0)**	1.11	0.30-409	0.871
**Stepwise regression analysis (Forward selection and backward elimination method)**			
**Metastasis** (Yes vs. No)	**2.72**	**1.44-5.14**	**0.002**

Dysphagia (Difficulty in swallowing) grade: 1-to solids, 2-to soft solids, 3-to liquids, 4-absolute; Significant values are shown in bold

### Gender specific association of inflammatory gene polymorphisms

We further stratified our overall group of subjects based on gender and found female specific increased risk of ESCC with TNFA -308 GA+AA genotype (OR = 3.25, 95% CI = 1.22-8.66, P =0.019) even after FDR test (q value = 0.069) (Table 5). However, *NFKBIA* -826 CT and CT+TT genotypes conferred 50% reduced risk of ESCC in female subjects (OR = 0.48, 95% CI = 0.25-0.91, P = 0.025, q = 0.045 and OR = 0.52, 95% CI = 0.28-0.97, P = 0.038, q = 0.057) (Table 5). No male-specific association of the studied gene polymorphisms was observed.

**Table 5:** Gender specific association of selected polymorphism with risk of ESCC.

**Genotypes**	**Males**			**Females**			
	**Controls N (%)**	**Patients N (%)**	OR* (95% CI ) P value	**Controls N (%)**	**Patients N (%)**	OR* (95% CI ) P value	
***TNFA* -308 G>A polymorphism**							
GG	185 (83.7)	163 (77.3)	Reference	83 (92.2)	64 (81.0)		Reference
GA	35 (15.8)	48 (22.7)	1.55 (0.95-2.51)0.079	7 (7.8)	14 (17.7)		**3.06 (1.14-8.27) 0.027^1^**
AA	1 (0.5)	0 (0)	NC	0	1 (1.3)		NC
GA+AA	36 (16.3)	48 (22.7)	1.50 (0.93-2.43) 0.098	7 (7.8)	15 (19.0)		**3.25 (1.22-8.66) 0.019^2^**
***NFKB1* -94 ATTG ins/del polymorphism**							
ATTG_1/_ATTG_1_	16 (7.2)	17 (8.1)	Reference	6 (6.7)	10 (12.7)		Reference
ATTG_1_/ATTG_2_	94 (42.5)	102 (48.3)	1.04 (0.50-2.17) 0.924	35 (38.9)	30 (38.0)		0.53 (0.17-1.63) 0.264
ATTG_2_/ATTG_2_	111 (50.2)	92 (43.6)	0.79 (0.38-1.66) 0.533	49 (54.4)	39 (49.4)		0.50 (0.16-1.49) 0.212
ATTG_1_/ATTG_2_+ ATTG_2_/ATTG_2_	205 (92.8)	194 (91.9)	0.90 (0.44-1.84) 0.780	84 (93.3)	69 (87.3)		0.51 (0.17-1.48) 0.214
**NFKBIA -826 C>T polymorphism**							
CC	112 (50.7)	99 (46.9)	Reference	37 (41.1)	46 (58.2)		Reference
CT	94 (42.5)	95 (45.0)	1.14 (0.77-1.68) 0.527	47 (52.2)	27 (34.2)		**0.48 (0.25-0.91) 0.025^3^**
TT	15 (6.8)	17 (8.1)	1.28 (0.61-2.71) 0.511	6 (6.7)	6 (7.6)		0.86 (0.26-2.93) 0.815
CT+TT	109 (49.3)	112 (53.1)	1.16 (0.79-1.69) 0.453	53 (58.9)	33 (41.8)		**0.52 (0.28-0.97) 0.038^4^**
***NFKBIA* 3’UTR A>G polymorphism**							
AA	38 (17.2)	51 (24.2)	Reference	21 (23.3)	20 (25.3)		Reference
AG	117 (52.9)	104 (49.3)	0.65 (0.40-1.07) 0.092	48 (53.4)	36 (45.6)		0.76 (0.36-1.63) 0.487
GG	66 (29.6)	56 (26.5)	0.62 (0.35-1.07) 0.087	21 (23.3)	23 (29.1)		1.21 (0.51-2.86) 0.670
AG+GG	183 (82.8)	160 (75.8)	0.64 (0.40 -1.03) 0.064	69 (76.7)	59 (74.7)		0.90 (0.44-1.82) 0.760

^1^, 0.069^2^, 0.045^3^, 0.057^4^; significant values are shown in bold; NC = not calculated^*^ age and gender adjusted odds ratio; FDR q value = 0.108

Association of the TNFA-308 G>A, *NFKB1* -94ATTG ins/del and *NFKBIA* polymorphisms with clinical characteristics (tumour location and regional lymph node involvement) of ESCC

Association of the polymorphisms with tumour location showed significant reduced risk of upper and lower third esophageal tumours with NFKBIA -826 CT+TT and 3’UTR AG+GG genotype respectively (OR = 0.41, 95% CI = 0.21-0.80, P = 0.009, q = 0.019 and OR = 0.52, 95% CI = 0.29-0.93, P = 0.028, q = 0.072 respectively) (Table 6). *TNFA* -308 or *NFKB1* -94 ins/del polymorphisms did not modulate any site-specific risk of ESCC tumours. We also analysed the role of the genetic variants with regional lymph node involvement and found ESSC patients with *TNFA* -308 GA or GA+AA genotypes were at two fold increased risk of regional lymph node involvement (GA vs. AA: OR =2.16, 95% CI = 1.27-3.67, P = 0.004, q =0.011; GA +AA vs. AA: OR = 2.18, 95% CI = 1.29-3.68, P = 0.003, q = 0.008) (Table 6). 

**Table 6:** Association of selected polymorphisms with clinical characteristics (tumour location and regional lymph node involvement) and risk of ESCC.

**Genotypes**	**Controls N (%)**	**Upper N (%)**	OR* (95% CI ) P value	**Middle N (%)**	OR* (95% CI ) P value	**Lower N (%)**	OR* (95% CI ) P value	**Regional lymph node involvement N (%)**	OR* (95% CI ) P value
***TNFA* -308 G>A polymorphism**									
GG	268 (86.2)	35 (77.8)	Reference	136 (79.1)	Reference	56 (76.7)	Reference	89 (74.2)	Reference
GA	42 (13.5)	10 (22.2)	1.83 (0.83-4.01) 0.132	35 (20.3)	1.62 (0.98-2.66) 0.058	17 (23.3)	1.88 (1.00-3.56) 0.051	30 (25.0)	**2.16 (1.27-3.67) 0.004^1^**
AA	1 (0.3)	0 (0)	NC	1 (0.6)	1.90 (0.12-30.67) 0.651	0 (0)	NC	1 (0.8)	3.00(0.19-48.59) 0.439
GA+AA	43 (13.8)	10 (22.2)	1.79(0.82-3.92) 0.146	36 (20.9)	1.62 (0.99-2.65) 0.053	17 (23.3)	1.84 (0.98-3.47) 0.60	31 (25.8)	**2.18 (1.29-3.68**) 0.003^2^
***NFKB1* -94ATTG ins/ del polymorphism**									
ATTG_1/_ATTG_1_	22 (7.1)	5 (11.1)	Reference	15 (8.7)	Reference	7 (9.6)	Reference	9 (7.5)	Reference
ATTG_1_/ATTG_2_	129 (41.5)	22 (48.9)	0.75 (0.26-2.19) 0.599	82 (47.7)	0.93 (0.46-1.90) 0.840	28 (38.4)	0.69 (0.28-1.77) 0.439	55 (45.8)	1.04 (0.45-2.41) 0.926
ATTG_2_/ATTG_2_	160 (51.4)	18 (40.0)	0.49 (0.16-1.45) 0.486	75 (43.6)	0.68 (0.33-1.39) 0.292	38 (52.1)	0.76 (0.30-1.91) 0.559	56 (46.7)	0.85 (0.37-1.97) 0.710
ATTG_1_/ATTG_2_ + ATTG_2_/ ATTG_2_	289 (92.9)	40 (88.9)	0.60 (0.22-1.69) 0.334	157 (91.3)	0.79 (0.40-1.57) 0.505	66 (90.4)	0.73 (0.30-1.78) 0.486	111 (92.5)	0.94 (0.42-2.10) 0.874
**NFKBIA -826 C>T polymorphism**									
CC	149 (47.9)	31 (68.9)	Reference	76 (44.2)	Reference	38 (52.1)	Reference	57 (47.5)	Reference
CT	141 (45.3)	11 (24.4)	**0.37 (0.18-0.76) 0.007^3^**	80 (46.5)	1.11 (0.75-1.64) 0.604	31 (42.5)	0.88 (0.52-1.49) 0.630	52 (43.3)	0.97 (0.62-1.50) 0.876
TT	21 (6.8)	3 (6.7)	0.68 (0.19-2.43) 0.553	16 (9.3)	1.53 (0.75-3.10) 0.241	4(5.4)	0.74 (0.24-2.28) 0.594	11 (9.2)	1.37 (0.62-3.03) 0.434
CT+TT	162 (52.1)	14 (31.1)	**0.41 (0.21-0.80) 0.009^4^**	96 (55.8)	1.16 (0.80-1.69) 0.431	35 (57.9)	0.86 (0.51-1.43) 0.561	63 (52.5)	1.02(0.67-1.55) 0.934
***NFKBIA 3’UTR A>G* polymorphism**									
AA	59 (19.0)	12 (26.7)	Reference	37 (21.5)	Reference	22 (30.1)	Reference	24 (20.0)	Reference
AG	165 (53.1)	23 (51.1)	0.67 (0.31-1.43) 0.299	81 (47.1)	0.77 (0.47-1.26) 0.298	36 (49.3)	0.57 (0.31-1.05) 0.070	68 (56.7)	1.02(0.59-1.77) 0.948
GG	87 (28.0)	10 (22.2)	0.54 (0.22-1.34) 0.183	54 (31.4)	0.97 (0.57-1.67) 0.922	15 (20.5)	**0.43 (0.21-0.91) 0.027^5^**	28 (23.3)	0.80 (0.42-1.51) 0.486
AG+GG	252 (81.0)	33(73.3)	0.62 (0.30-1.29) 0.201	135 (78.5)	0.84 (0.53-1.34) 0.461	51 (69.9)	**0.52 (0.29-0.93) 0.028^6^**	96 (80.0)	0.94 (0.55-1.61) 0.827

^1^, 0.008^2^, 0.014^3^, 0.019^4^, 0.077^5^, 0.072^6^; significant values are shown in bold; NC = not calculated^*^ age and gender adjusted odds ratio; FDR q value = 0.011

### Interaction of the polymorphisms with environmental risk factors

A case only analysis was performed to evaluate interaction of the polymorphisms with environmental risk factors, however, no significant modulation in risk of ESCC was found in tobacco users, smokers and alcohol users (Data not shown). 

### Linkage disequilibrium and haplotype analysis of NFKBIA polymorphisms

Linkage disequilibrium (LD) analysis showed that NFKBIA -826 C>T and 3’UTR A>G polymorphisms were in moderate LD in controls as well as in patients (D’ = 0.400, χ^2^ = 33.71, P < 0.001 and D’ = 0.609, χ^2^ = 84.65, P < 0.001 respectively). A total of four haplotypes were observed in the subjects. The frequency of C_-826_G_3_’_UTR_ and T_-826_A_3_’_UTR_ haplotypes were significantly lower in cases (27.9% and 5.5% respectively) than controls (33.1% and 8.0% respectively). Therefore, C_-826_G_3_’_UTR_ and T_-826_A_3_’_UTR_ haplotypes were associated with reduced risk of ESCC compared to C_-826_A_3_’_UTR_ haplotype (OR = 0.73, 95% CI = 0.56-0.96, P = 0.025 and OR = 0.60, 95% CI = 0.37-0.96, P = 0.034) (Table 7).

**Table 7:** Association of *NFKBIA* haplotypes with the risk of ESCC.

**Haplotypes**	**Controls N (%)**	**Patients N (%)**	**OR (95% CI ) P value**
C_-826_ A_3'__UTR_	233 (37.5)	250 (43.1)	Reference
C_-826_ G_3'__UTR_	206 (33.1)	162 (27.9)	**0.73 (0.56 - 0.96) 0.025**
T_-826_ G_3'__UTR_	133 (21.4)	136 (23.5)	0.95 (0.71 - 1.28) 0.752
T_-826_ A_3'_'_UTR_	50 (8.0)	32 (5.5)	**0.60 (0.37 - 0.96) 0.034**

Significant values are shown in bold

### Combined risk genotype analysis of TNFA-308 G>A, *NFKB1* -94ATTG ins/del and *NFKBIA* (-826 C>T and 3’**UTR A**>G) polymorphisms

We also performed combined risk genotype analysis to study the effect of risk genotypes of all four polymorphisms on susceptibility to ESCC. For this, we pooled risk genotype (OR >1) of all four SNPs into new variable according to the number of risk genotypes, ranging from 0-4 (in case of protective association, we reversed the reference group). A significant dose dependent risk of ESCC was observed with combined risk genotype of all four SNPs (P_trend_ = 0.001). Moreover, in dichotomized analysis, patients with 1-3 risk genotype had 1.67 fold higher risk of ESCC compared to patients with ‘0’ risk genotype (OR =1.67, 95% CI = 1.21-2.31, P =0.002, q value = 0.005) (Table 8). Role of combined risk genotype in the prognosis of ESCC patients was also analyzed. However, no significant difference in median survival was found among patients carrying ‘0’, ‘1’ ‘2’ or ‘3’risk genotypes (Log rank P value = 0.882). Cox regression analysis also did not show any significant hazard of death with any of the risk genotypes i.e., ‘1’ ‘2’ or ‘3’risk genotypes (Data not shown). Furthermore, there was no effect of dichotomized risk genotype (1-3 vs. 0 risk genotype) on survival outcome of ESCC patients (Table 8).

**Table 8:** Role of combined risk genotypes of selected gene polymorphisms in the risk and prognosis of esophageal cancer.

**Risk group**	**Controls N (%)**		**Cases N (%)**	OR*(95% CI) P value
**Combined Genotype**				
0	186 (59.8)		137 (47.2)	Reference
1	106 (34.1)		123 (42.4)	**1.58 (1.12-2.23) 0.009**
2	18 (5.8)		29 (10.0)	**2.22 (1.18-4.17) 0.013**
3	1 (0.3)		1 (0.3)	1.34 (0.08-21.70) 0.836
P for trend	**0.00123**			
**Dichotomized**				
0	186 (59.8)		137 (47.2)	Reference
1-3	125 (40.2)		153 (52.8)	**1.67 (1.21-2.31) 0.002^1^**
**Dichotomized risk genotype**	**N (%)**	**Median survival (in months)**	**Log Rank P value**	**HR (95% CI) P value**
0	80 (49.4)	18.80	0.528	Reference
1-3	82 (50.6)	15.00		0.88 (0.56-1.38) 0.877

^1^, significant values are shown in bold^*^ age and gender adjusted odds ratio; FDR q value = 0.005

### Multi-dimensionality reduction (MDR) analysis

MDR analysis showed that *TNFA* -308 polymorphism was the best one factor model and *TNFA* -308 and *NFKBIA* 3’UTR were the best model for two factors and *TNFA*-308, *NFKBIA* -826, *NFKBIA* 3'UTR polymorphisms as best model for three factors. However, P value of permutation testing was significant only for three factor model (P value = 0.006, 0.264 and 0.214 for three, two and one factor model respectively) (Table S2). Thus, three factor model *TNFA*-308, *NFKBIA*-826, *NFKBIA* 3’UTR with testing accuracy of 0.57 and 10/10 CVC is better predictor risk of ESCC compared to one/two factors. 

## Discussion

Inter-individual variation in ESCC may be partly attributed to genetic variants in inflammatory and immune-responsive genes [38,39]. In the present study, we investigated the role of common polymorphic variants in *TNFA*, *NFKB1* and *NFKBIA* (the major mediators of inflammatory and immune response in malignancy) with risk and prognosis of ESCC in northern Indian population. We observed that *TNFA*-308 G>A polymorphism was associated with enhanced risk of ESCC especially in females and in patients with regional lymph node involvements. No independent role of either *NFKB1* -94ATTG ins/del or *NFKBIA* (-826 C>*T* and 3’UTR A>G) polymorphisms in ESCC susceptibility was found, however, two haplotypes of *NFKBIA* polymorphisms (C_-826_ G_3_’_UTR_ and T_-826_ A_3_’_UTR)_ seem to have protective role in ESCC. MDR analysis showed *TNFA* -308, *NFKBIA* -826 and *NFKBIA* 3’UTR polymorphisms as better predictor for risk of ESCC. Moreover, when we pooled risk genotype of all the polymorphisms, a significant increased risk of ESCC was observed in subjects with ≥1 risk genotypes compared to subjects with ‘0’ risk genotype. 

The MAF of *TNFA* -308 G>A polymorphism in present study was 7.1% which is similar to Hapmap Guajarati Indian (GIH) and Han Chinese (CHB) population (http//hapmap.ncbi.nlm.nih.gov/). We found a significant 1.7 fold increased risk of ESCC with *TNFA* -308 GA genotype and A allele in comparison to GG genotype to G allele respectively. Literature suggests strong association of *TNFA* -308 A allele with MHC haplotype HLA-A1-B8-DR3, which is in turn linked with increased production of TNF-α [40,41]. Also, *TNFA* -308A allele is stronger transcriptional activator than G allele and production of TNF-α was reported to be higher in monocyte culture of individuals with *TNFA*-308 GA compared to that with GG genotype [42,43]. Thus, increased risk of ESCC with *TNFA* -308 GA genotype and A allele may be due to higher TNF-α production, which promotes malignant processes through induction of premalignant chemokines, angiogenic mediators, reactive oxygen intermediates and inflammatory mediators [4]. In contrast to our finding, two previous studies did not find independent association of *TNFA* -308 G>A polymorphism with susceptibility to gastro-esophageal cancer or ESCC [14,15]. However, their results should be interpreted cautiously as *TNFA*-308 polymorphism was not in HWE in controls in these studies (P_HWE_ were less than 0.05). For this reason, we could not perform meta-analysis of *TNFA* -308 polymorphism in esophageal cancer as one of exclusion criteria for studies in meta-analysis is disagreement of polymorphism with HWE in controls. 

 NF-κB is a major transcription regulator of immune response, apoptosis and cell-cycle genes [18]. A four base pair deletion polymorphism in *NFKB1* i.e., -94ATTG del is extensively explored in various cancers. The deletion allele of the polymorphism abolishes binding site for nuclear protein resulting in reduced promoter activity [44]. Although, prior studies have shown significant association of the *NFKB1* polymorphism with carcinoma of urinary bladder, prostate, cervix and nasopharynx [33,45–47], no role of the polymorphism in ESCC susceptibility was found in the present study. Similar to our study, Riemann et al. did not find association of the polymorphism with bladder cancer, colorectal cancer, renal cell carcinoma and B cell chronic lymphocytic leukemia in German subjects [48,49]. The different findings of *NFKB1* -94ATTG del polymorphism may be due to its cancer specific or population specific nature of association. We also examined association of two functional polymorphisms in *NFKBIA* (-826C>T and 3’UTRA>G) with ESCC susceptibility, however no significant role in ESCC was observed. 

Univariate survival analysis through Kaplan Meier test and Cox regression showed no difference in median survival or death hazard of ESCC patients with any of the selected gene polymorphism. Also, in multivariate analysis, no effect of studied gene polymorphisms in prognosis of ESCC was found. However, presence of metastasis was associated with worse survival outcome of ESCC patients in multivariate analysis, which is well known fact. A previous study showed that *TNFA*-308 AA genotype was associated with poor prognosis of tumour at gastro-esophageal (GE) junction study [15]. This could be due to entirely different etiology of tumour located at GE junction compared to esophageal squamous cell carcinoma. The lack of prognostic role of the selected gene polymorphisms in ESCC patients in the present study may be due to small number of alive cases (15.4%) compared to dead or lost to follow up cases (84.6%) or short median follow-up. 

Stratification based on gender showed that *TNFA-*308 GA was associated with increased risk of ESCC in females, in contrast, *NFKBIA* -826 CT genotype conferred female-specific decreased risk of ESCC. These is sexual dimorphism in immune/inflammatory response and females exhibits more vigorous cellular/ humoral immune reactions compared to males [50]. Also, female hormone progesterone was found to increases TNF-α secretion in activated monocytes [51]. So, female specific association of *TNFA-*308 A allele may explained by higher TNF-α level in females leading to aggressive inflammatory response. However, these findings (female specific associations) need to be confirmed in larger cohort and should be interpreted cautiously due to low number of female patients in the present study.

 ESCC shows considerable heterogeneity in clinical phenotypes and different genetic factors/pathways are implicated in development of specific esophageal tumour phenotypes [52,53]. In line with this, NFKBIA -826 CT+TT and 3’UTR AG+GG genotypes seem to be associated with lower risk of upper and lower third esophageal tumour respectively. Furthermore, *TNFA*-308 GA genotype conferred significant increased risk of regional lymph node involvement in ESCC patients. Similar to this, Kim et al. had shown association of the *TNFA* -308 polymorphism with lymph node metastasis in gastric cancer patients [54]. 

Regional lymph node involvement usually associated with poor clinical outcome of the cancer patients. However, in the present study no modification in prognosis of esophageal cancer patients was observed with lymph node status. This may be due to low number (64/162, 39.5%) of followed up patients had regional lymph node involvement. So, despite association *TNFA*-308 GA with regional lymph node involvement in ESCC patients, no role of *TNFA*-308 polymorphism in clinical outcome of ESCC patients was found.

We next carried out the haplotype analysis of two *NFKBIA* polymorphisms which showed significant decreased risk of ESCC with C_-826_G_3_’_UTR_ and T-_826_A_3_’_UTR_ haplotypes. Bioinformatics analysis showed that NFKBIA -826 C>T polymorphism may affect binding of transcription factor like *BRAC*1/2, *TBP* and *MYB*, while 3’UTRA>G polymorphism may affect binding of mir-196a (www.SNPinfo.com). Few studies have also examined the effect of *NFKBIA* 3’UTRA>G polymorphism on expression of the protein, however, finding are inconsistent. While, the expression of *NFKBIA* was reported to be higher in peri-tumour tissues from colorectal cancer patients with 3’UTR AA+AG genotypes than those with 3’UTR GG genotype [55], another study did not find difference in expression of *NFKBIA* in melanoma patients with different 3’UTR genotypes [56]. Data demonstrating exact functional role of *NFKBIA* -826 and 3’UTR polymorphisms in ESCC are lacking, however, it is reasonable to assume that differential expression of IκB due to *NFKBIA* specific haplotypes may result in different NF-κB activation, further leading to differential risk for ESCC.

Cancer is a multi-genic disease in which single SNP may only have a modest independent effect on disease phenotype and multiple SNPs may provide a more accurate representation of the risk. So we carried a high order interaction analysis to find out the best predictive model for the risk of ESCC. A three factors model *TNFA*-308, *NFKBIA* -826, *NFKBIA* 3’UTR polymorphisms was predicted to be best model with maximum testing accuracy and CV consistency. Furthermore, when we pooled the risk genotype (with OR>1) of all the polymorphisms, significant increased risk of ESCC of was observed with combined risk genotype in dose responsive manner. Also, individuals with 1-3 risk genotypes were found to have higher risk of ESCC compared to those with ‘0’ risk genotype. However, no role of combined risk genotype on survival outcome of ESCC patients was found. These findings suggest the joint effect of studied gene polymorphisms in susceptibility but not in prognosis of ESCC. Interaction of selected polymorphisms with environmental risk factors were also analyzed, which did not show any significant outcome. This implies that there may be other classes of genes which might show interaction with environmental risk factor for developing risk of ESCC in northern Indian population.

The present study has several strengths like well defined set of cases and controls, agreement of genotypic data with HWE in controls, consistencies of MAF of polymorphisms with Hapmap GIH data and adoption of stringent quality control measures. Limitation of study is low sample size in subgroups, absence of qualitative environmental exposure data in cases and short median follow-up in survival analysis. 

In summary, our results suggest independent role of *TNFA* -308 G>A polymorphism and combined effect of *TNFA* and *NFKBIA* gene polymorphisms in susceptibility of ESCC in northern Indian population. However, none of the genetic variants seem to have implications in prognosis of ESCC. 

## Supporting Information

Table S1 **Details of genotyping methods of TNFA-308 G>A, NFKB1 -94ATTG ins/del and NFKBIA (-826 C>T and 3’UTR A>G) polymorphisms.**
(DOCX)

Table S2 **Multi Dimensionality Reduction (MDR) analysis of selected gene polymorphisms.**
(DOCX)
