# Supplementary material for: Association of Common Polymorphisms in TNFA, NFkB1 and NFKBIA with Risk and Prognosis of Esophageal Squamous Cell Carcinoma
Source: PLoS One. 2013 Dec 4;8(12):e81999. doi: 10.1371/journal.pone.0081999 (PMC3852749; doi:10.1371/journal.pone.0081999)
Supplement: Table S1 — Details of genotyping methods of TNFA-308 G>A, NFKB1 -94ATTG ins/del and NFKBIA (-826 C>T and 3’UTR A>G) polymorphisms. (DOCX) [file pone.0081999.s001.docx]

**Table S1** Details of genotyping methods of *TNFA*-308 G>A, *NFKB1* -94ATTG ins/del and *NFKBIA* (-826 C>T and 3’UTR A>G) polymorphisms

| **Polymorphisms** | **Genotyping Methods** | **Primer / Probe Sequences** | **Genotyping pattern** | **References** |
| --- | --- | --- | --- | --- |
| ***TNFA*-308 G>A** | ARMS PCR | F1:TGGAGGCAATAGGTTTTGAGGGGCAGGA  R1:TAGGACCCTGGAGGCTGAACCCCGTACC  F2:ACCCAAACACAGGCCTCAGGACTCAACA  R2:AGTTGGGGACACGCAAGCATGAAGGATA | G allele: 224 bp  A allele: 154 bp  Common band: 323 bp | [32] |
| ***NFKB1* -94ATTG ins/del** | PCR | F: TGGACCGCATGACTCTATCA  R: GGCTCTGGCTTCCTAGCAG | ATTG_1_/ATTG_1_/: 154 bp  ATTG_1_/ ATTG_2:_ 158 bp and 154 bp  ATTG_2_/ ATTG_2:_ 158 bp | [33] |
| ***NFKBIA* -826 C>T** | TaqMan assay | FAM labeled: TGGTGGTTGTGGATACCTTGC AATAGCAGAGTAGCTATTGTGTTCATAAGT  VIC labeled: TGGTGGTTGTGGATACCTTG CAATAACAGAGTAGCTATTGTGTTCATAAGT | - | Applied Biosystems Inc, Foster City, USA |
| ***NFKBIA* 3’-UTR A>G** | PCR RFLP (Restriction enzyme- *Hae III*) | F: GGCTGAAAGAACACTTG  R: GTACACCATTTACAGGG | AA: 424 bp  AG: 316 bp. 108 bp and 424 bp  GG: 316 bp and 108 bp | [34] |

F: forward primer, R: reverse primer
