# Supplementary material for: Association of Common Polymorphisms in TNFA, NFkB1 and NFKBIA with Risk and Prognosis of Esophageal Squamous Cell Carcinoma
Source: PLoS One. 2013 Dec 4;8(12):e81999. doi: 10.1371/journal.pone.0081999 (PMC3852749; doi:10.1371/journal.pone.0081999)
Supplement: Table S2 — Multi Dimensionality Reduction (MDR) analysis of selected gene polymorphisms. (DOCX) [file pone.0081999.s002.docx]

**Table S2** Multi Dimensionality Reduction (MDR) analysis of selected gene polymorphisms

| **Number of interacting loci** | **Best Model** | **Testing accuracy** | **Cross-validation consistency (CVC)** | **P value for Permutation** |
| --- | --- | --- | --- | --- |
| 1 | *TNFA*-308 | 0.5395 | 10/10 | 0.2140 |
| 2 | *TNFA*-308, *NFKBIA* 3’UTR | 0.5358 | 7/10 | 0.2640 |
| 3 | *TNFA-308*, *NFKBIA*-826*, NFKBIA* 3’UTR* | 0.5723 | 10/10 | **0.0060** |

* Best model was selected based on maximum testing accuracy and CVC; significant values are shown in bold
